# Anabolic Steroids-Driven Regulation of Porcine Ovarian Putative Stem Cells Favors the Onset of Their Neoplastic Transformation

**DOI:** 10.3390/ijms222111800

**Published:** 2021-10-30

**Authors:** Gabriela Gorczyca, Kamil Wartalski, Jerzy Wiater, Marcin Samiec, Zbigniew Tabarowski, Małgorzata Duda

**Affiliations:** 1 Department of Endocrinology, Faculty of Biology, Institute of Zoology and Biomedical Research, Jagiellonian University in Krakow, Gronostajowa 9 Street, 30-387 Krakow, Poland; gabriela.gorczyca@uj.edu.pl; 2 Department of Histology, Faculty of Medicine, Jagiellonian University Medical College, Kopernika 7 Street, 31-034 Krakow, Poland; kamil.wartalski@uj.edu.pl (K.W.); jerzy.wiater@uj.edu.pl (J.W.); 3 Department of Reproductive Biotechnology and Cryoconservation, National Research Institute of Animal Production, Krakowska 1 Street, 32-083 Balice near Kraków, Poland; 4 Department of Experimental Hematology, Faculty of Biology, Institute of Zoology and Biomedical Research, Jagiellonian University in Krakow, Gronostajowa 9 Street, 30-387 Krakow, Poland; zbigniew.tabarowski@uj.edu.pl

**Keywords:** pig, ovary, putative stem cells, nandrolone, boldenone, neoplastic transformation

## Abstract

Nandrolone (Ndn) and boldenone (Bdn), the synthetic testosterone analogues with strong anabolic effects, despite being recognized as potentially carcinogenic compounds, are commonly abused by athletes and bodybuilders, which includes women, worldwide. This study tested the hypothesis that different doses of Ndn and Bdn can initiate neoplastic transformation of porcine ovarian putative stem cells (poPSCs). Immunomagnetically isolated poPSCs were expanded ex vivo in the presence of Ndn or Bdn, for 7 and 14 days. Results show that pharmacological doses of both Ndn and Bdn, already after 7 days of poPSCs culture, caused a significant increase of selected, stemness-related markers of cancer cells: CD44 and CD133. Notably, Ndn also negatively affected poPSCs growth not only by suppressing their proliferation and mitochondrial respiration but also by inducing apoptosis. This observation shows, for the first time, that chronic exposure to Ndn or Bdn represents a precondition that might enhance risk of poPSCs neoplastic transformation. These studies carried out to accomplish detailed molecular characterization of the ex vivo expanded poPSCs and their potentially cancerous derivatives (PCDs) might be helpful to determine their suitability as nuclear donor cells (NDCs) for further investigations focused on cloning by somatic cell nuclear transfer (SCNT). Such investigations might also be indispensable to estimate the capabilities of nuclear genomes inherited from poPSCs and their PCDs to be epigenetically reprogrammed (dedifferentiated) in cloned pig embryos generated by SCNT. This might open up new possibilities for biomedical research aimed at more comprehensively recognizing genetic and epigenetic mechanisms underlying not only tumorigenesis but also reversal/retardation of pro-tumorigenic intracellular events.

## 1. Introduction

Individual tumors consist of mixed cell populations that differ in function, morphology, and molecular signatures. Of these, only a small subset of tumor cells is capable to initiate and sustain tumor growth. These cells were termed cancer stem cells (CSCs) [1,2]. On the basis of functional and molecular analysis of CSCs isolated from many organs, it was confirmed that they display stem cell-like characteristics. These are self-renewal, multi-lineage differentiation and expression of stemness-related markers [3,4]. Some of these features have been even confirmed by the analysis of single cells [5]. Based on the above, it is believed that CSCs may play a crucial role in disease recurrence after treatment and remission. Similar to ESCs and ASCs, CSCs express markers that are not expressed by normal somatic cells and are thus thought to contribute towards a ‘stemness’ phenotype. Various specific markers (clusters of differentiation), including CD44 and CD133, have been employed for the isolation and characterization of ovarian CSCs from ovarian cancer cell lines and patients’ tumors [6,7,8,9]. Importantly, CD44 and CD133 are the most widely used markers in CSCs research and they are also therapeutic targets in cancers [10].

Cluster of differentiation 44 (CD44), a transmembrane glycoprotein, is expressed normally by both fetal and adult hematopoietic stem cells. Upon binding to hyaluronic acid (HA), its primary ligand, CD44, mediates cell division, migration, adhesion, and signaling [11]. CD44 is highly expressed in many types of cancers including breast, prostate, and ovarian cancers [12,13]. Zhang et al. [14] have shown that ovarian cancer initiating cells (OCICs) express both CD44 and CD117. In turn, Bourguignon et al. [15] proved the dependence between CD44 receptor and pluripotency proteins, mainly NANOG, in human ovarian cancer cells line. Binding to HA, CD44 receptor promotes association of the NANOG protein with CD44, followed by NANOG activation and expression of pluripotent stem cell regulators, e.g., Rex1 and Sox2. Thus, CD44 supports the differentiation and proliferation of neoplastic cells [15]. CD133, another transmembrane glycoprotein, is normally expressed in hematopoietic stem cells, endothelial progenitor cells, and neuronal and glial stem cells [16]. CD133 is involved in cell growth and development [17]. Almost all tumor types, including ovarian ones, can be detected on the basis of CD133 expression. Interestingly, in ovarian tumors both epitopes, i.e., CD133-1 and CD133-2, have been detected [18]. What is more, CD133 is highly specific for rare and phenotypically distinct population of CSCs occurring in solid ovarian tumors [19]. Moreover, CD133-positive neoplastic cells have been found to display such stem cell-related attributes as self-renewal, differentiation, and tumor formation in the NOD-SCID mouse model. After injection into immune-compromised mice, CD133-positive neoplastic cells also exhibit chemo- and radio-resistance [20], which makes CD133 a potential anti-cancer therapeutic target [21].

Anabolic androgenic steroids (AAS) are substances synthesized from testosterone or one of its derivatives, with anabolic or androgenic properties, depending on the target tissue [22,23]. AAS exert their effects by binding to androgen receptors (ARs), thus mimicking or blocking their action, by altering endogenous hormone levels or by modifying hormone receptor turn over [24]. Importantly, the International Agency for Research on Cancer classified AAS to the group of potentially carcinogenic compounds for humans. These compounds may exert both genotoxic and cytotoxic effects, which can lead to the formation of a tumor [25]. AAS, including nandrolone (Ndn) and boldenone (Bdn), are rapidly becoming a widespread group of drugs used both clinically and illicitly. The illicit use of AAS is diffused among adolescents and bodybuilders because of their anabolic proprieties and their capacity to increase tolerance to exercise. Moreover, despite the regulations (The European Community banned the use of anabolics in Europe by means of laws 96/22/EC and 96/23/EC), these compounds are still widely used in fodder for farm animals, and their metabolites end up in the environment with their urine. Ndn is an androgen receptor agonist but also a potent progestogen [26]. In medicine, it is used in convalescence after debilitating diseases and to improve the body structure and physical condition of the body [27,28]. It is also known that the pharmacological dose of Ndn slows down cell growth, inhibits mitochondrial respiration, inhibits respiratory chain complexes I and III, and increases the production of mitochondrial reactive oxygen species (ROS). Whereas chronic administration of Ndn favors the maintenance of stem cells in various tissues but may increase the risk of their neoplastic transformation [29]. Bdn is also a dehydrated testosterone analog [26] and an androgen receptor agonist [28]. Bdn is frequently abused because it increases appetite, protein synthesis, nitrogen retention, and also stimulates the release of erythropoietin in the kidneys [30]. Although, the positive and deleterious effects promoted by AAS are well documented, none of the studies have focused on their effects on mechanisms responsible for the initiation of stem cells’ possible neoplastic transformation. 

Among various animal experimental models, pigs share many similarities with humans in the form of organ size, physiology, and functioning [31]. The limited ethical dilemmas and importantly, successful isolation of putative stem cells from the ovarian cortex (poPSCs) [32,33,34], make pigs the valuable experimental model for not only preclinical assessment for stem cell therapy but also for the purposes of somatic cell nuclear transfer (SCNT)-based cloning in mammals. The efforts undertaken to use poPSCs, which have been subjected to extrinsic anabolic steroid-dependent oncogenic transformation (carcinogenesis), as a completely new source of nuclear donor cells (NDCs) for future research focused on SCNT, have not yet been accomplished. The results of the studies that sought to examine the suitability of malignant neoplastic cells derived from cerebellum-specific medulloblastoma [35] and breast cancer [36] as the sources of NDCs, successfully promoting the in vitro and/or early in vivo development of murine cloned embryos, have already confirmed the improvements in epigenetic reprogrammability of such NDCs following reconstruction and activation of SCNT-derived mouse oocytes. Furthermore, investigations by Li et al. [35] and Shao et al. [36] have proved that NDCs stemming from metastatic cancers have irreversibly lost their pro-oncogenic genotypic attributes and pro-cancerous phenotypic traits due to efficient epigenetic reprogramming of their nuclear genomes in murine SCNT-derived oocytes and corresponding embryos. The identifying capabilities of cancerous tumor-derived NDCs to be epigenetically reprogrammed into normal (i.e., noncancerous) cell types could give rise to the development of a general strategy reliable and feasible for assessing the contribution of genetic and epigenetic factors to not only tumorigenesis but also cessation of pro-cancerous scenario of molecular pathways followed by onset and subsequent recapitulation of anti-oncogenic transformation. Thus, exploring the extent of epigenomic plasticity and reprogrammability that determine the incidence of reversal (abrogation) and cessation (suppression) of molecular events leading to pro-cancerous conversion of poPSCs, whose nuclear genomes have been used for generating porcine SCNT-derived embryos, seems to be an especially attractive research problem. Solving this problem might contribute to the increase in the efficiency of somatic cell cloning in pigs and other mammalian species. 

To the best of our knowledge, no studies about anabolic steroid-triggered immortalization of poPSCs via the activation of molecular pathways related to neoplastic transformation (neoplasia) have been reported so far. To meet this goal, we examined: (1) possible interactions of AAS with the AR in poPSCs and (2) amount and location of the most widely-used markers of CSCs: CD44 and CD133, both at mRNA and protein levels, after exposure to different doses of Ndn and Bdn in vitro. Additionally, we tested the effect of selected AAS on proliferation, viability, incidence of apoptotic events, and modulation of mitochondrial oxidative metabolism of poPSCs.

## 2. Results

### 2.1. poPSCs Cultured In Vitro with or without the Presence of Boldenone and Nandrolone Express the Androgen Receptor

**Figure 1:**
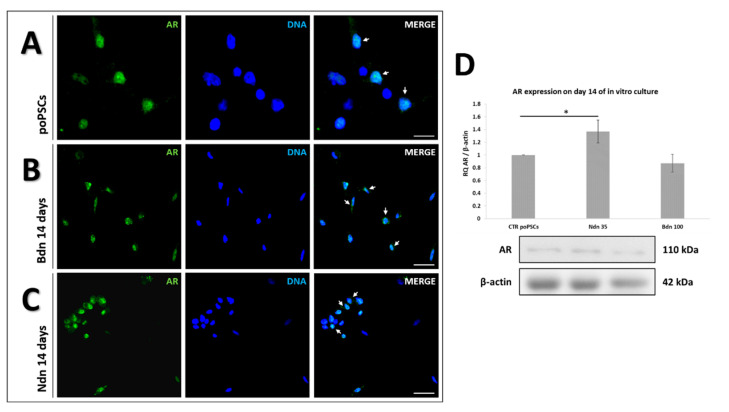
The presence and localization of AR in poPSCs cultured without the addition of anabolic steroids (**A**) and in poPSCs cultured for 14 days in the presence of boldenone at a dose of 100 µM (**B**) and nandrolone at a dose of 35 µM (**C**). Green signal-AlexaFluor 488 fluorescent dye, blue signal–DAPI; scale bars represent 50 µm in A and 100 µm in B and C. The immunofluorescence staining was repeated thrice; the figure shows the best representative micrographs selected from 3 replicates. Expression of AR at the level of total protein after 14-Day culture in the presence of nandrolone and boldenone (**D**). The graphs depict the relative abundances (RAs) noticed for AR obtained from measurements of the optical density of the bands representing a specific signal. Results represent the mean with *n* = 3 ± standard deviation (SD). Statistical analysis: homogeneity of variance—Levene’s test, normality of distribution-Shapiro–Wilk test, one-way ANOVA, Tukey’s post-hoc test, * *p* < 0.05.

Androgen receptor showed a specific nuclear localization (Figure 1) in both poPSCs cultured without anabolic steroid addition (line A) and in those cultured for 14 days with the addition of Bdn (line B) or Ndn (line C). The intense green color (white arrows) from the Alexa488 fluorochrome, which coincides with the blue signal from DAPI in the cell nucleus, indicates the nuclear localization of AR in poPSCs. The results indicate that these anabolic steroids do not disturb AR expression in poPSCs. They also provide an indirect demonstration that both nandrolone and boldenone bind to ARs and induce their activation, thus exerting the biological effects in poPSCs. After a 14-Day in vitro culture of poPSCs in the presence of nandrolone, a statistically significant (* *p* < 0.05) increase in ARs expression was observed. While the 14-Day exposure of poPSCs to boldenone caused a slight decrease in AR expression, it was not a statistically significant change.

### 2.2. Nandrolone and Boldenone Affect the Proliferation of poPCS after 14-Day In Vitro Culture

**Figure 2:**
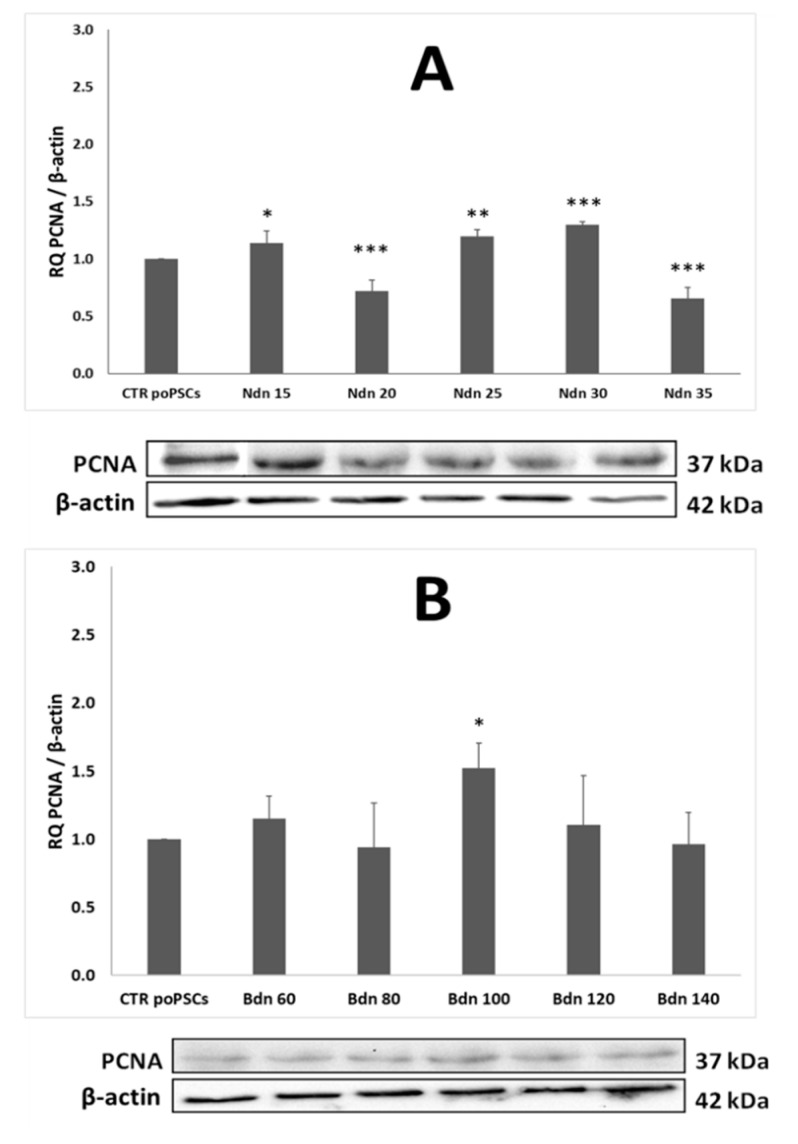
PCNA proliferation marker expression at the level of total protein isolated from poPSCs grown at different concentrations of nandrolone (**A**) and boldenone (**B**). The graphs show the relative content of PCNA protein obtained from measurements of the optical density of the bands representing a specific signal. Results represent the mean with *n* = 5 ± standard deviation (SD). Statistical analysis: homogeneity of variance—Levene’s test, normality of distribution—Shapiro–Wilk test, one-way ANOVA, and Duncan’s post-hoc test: part of the differences on the level of PCNA expression was statistically significant as follows: * *p* < 0.05; ** *p* < 0.01; *** *p* < 0.001. N 15-N 35: applied doses of nandrolone in concentrations ranging from 15 μM to 35 μM; B 60-B 140: doses of boldenone used in concentrations ranging from 60 μM to 140 μM, CTR poPSCs-control culture without the addition of anabolic steroids.

In order to test the effect of Ndn and Bdn on cell proliferation, poPSCs were treated for 14 days with these drugs at concentrations ranging from 15 to 35 μM and 60 to 140 μM for Ndn and Bdn, respectively. Lysates from poPSCs cultured in the presence of various concentrations of AAS were tested for the expression level of the PCNA proliferation marker against poPSCs cultured without these steroids. Figure 2 shows the results noticed for relative expression of proliferating cell nuclear antigen (PCNA), which is normalized to the reference protein of β-actin. Based on this, the treatment with 35 μM of nandrolone was chosen, since it caused a marked inhibition of cell growth still preserving cell viability. In turn, 100 μM of boldenone induced the opposite effect—a significant increase in the proliferation of poPSCs; thereby this dose was selected for further experiments.

### 2.3. Boldenone and Nandrolone Influence on poPSCs Viability, Cytotoxicity, and Apoptotic Activity

To further understand how the selected doses of both anabolic steroids (35 μM for nandrolone and 100 μM for boldenone) impact the viability, cytotoxicity, and caspase activation-related events in poPSCs during 14 days of their culture, the ApoTox-Glo triplex assay was performed. 

**Figure 3:**
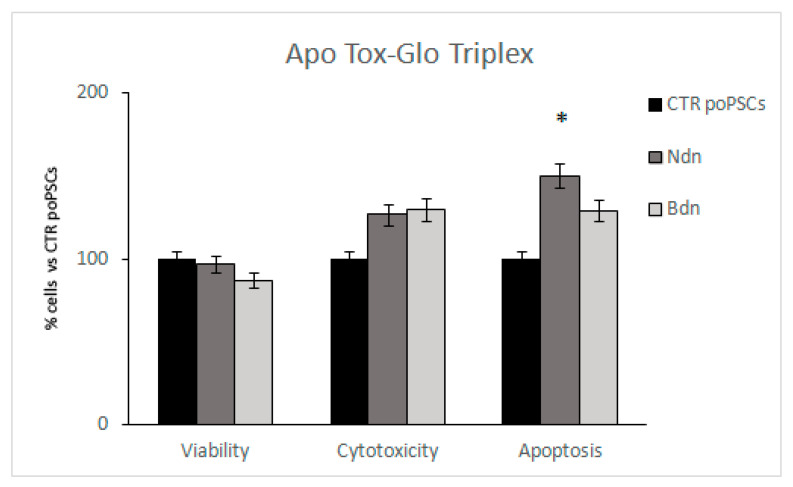
The effect of 14-Day treatment of poPSCs with either 35 μM of nandrolone or 100 μM of boldenone on cellular viability, cytotoxicity, and apoptotic cell death (estimated by ApoTox-Glo Triplex Assay). Results were expressed as percentages with the poPSCs control values (CTR) taken as 100%. The results represent the mean with *n* = 5 ± standard deviation (SD). Statistical analysis: homogeneity of variance—Levene’s test, normality of distribution—Shapiro–Wilk test, one-way ANOVA, Tukey’s post-hoc test, * *p* < 0.05.

Both nandrolone and boldenone have been found to insignificantly bias the viability and cytotoxicity among ex vivo-expanded poPSCs. In turn, 14-Day exposure of poPSCs to 35 μM of nandrolone induced their apoptosis (* *p* < 0.05) (Figure 3).

### 2.4. Nandrolone and Boldenone Trigger the Expression of Selected Cancer Stem Cells Markers: CD44 and CD133

**Figure 4:**
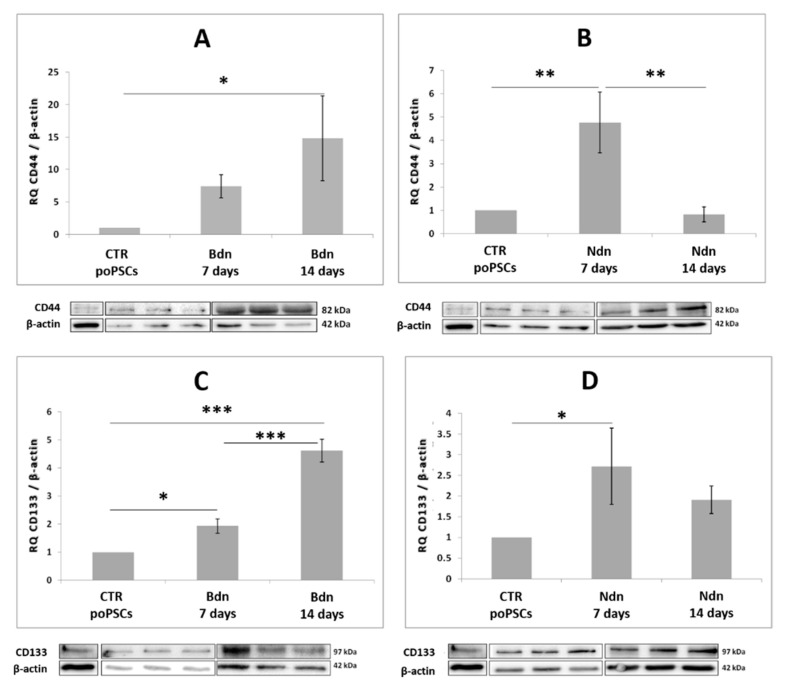
Expression of the protein markers CSCs: CD44 (**A**,**B**) and CD133 (**C**,**D**) at the level of total protein on day 7 and 14 of culture in the presence of boldenone (**A**,**C**) and nandrolone (**B**,**D**). The graphs show the relative content of CD44 and CD133 proteins obtained from measurements of the optical density of the bands representing a specific signal. Results represent the mean with *n* = 5 ± standard deviation (SD). Statistical analysis: homogeneity of variance—Levene’s test, normality of distribution—Shapiro–Wilk test, one-way ANOVA, Tukey’s post-hoc test, * *p* < 0.05; ** *p* < 0.01; *** *p* < 0.001.

In poPSCs cultured with nandrolone supplementation, an increase in the abundance of CD44 protein (** *p* < 0.01) was observed after 7 days of treatment. Next, a decrease to the value found in control poPSCs cultures was noted (Figure 4B). The profile of CD133 protein abundance in poPSCs cultured under the influence of nandrolone was very similar. After 7 days of poPSCs-nandrolone treatment, increase in CD133 abundance (* *p* < 0.05) was observed. Prolonged treatment of poPSCs with nandrolone for up to 14 days decreased the CD133 protein abundance to the value found in the poPSCs control cultures (Figure 4D).

A slightly different situation was observed in poPSCs cultured under the influence of boldenone. CD44 protein abundance significantly increased (* *p* < 0.05) in poPSCs cultured in vitro for 14 days in the presence of boldenone, while 7 days of exposure to boldenone induced its statistically insignificant increase (Figure 4A) when compared to that in the corresponding poPSCs control cultures. In turn, CD133 protein abundance was markedly increased in poPSCs cultured in the presence of boldenone for both 7- and 14-days (* *p* < 0.05 and *** *p* < 0.001, respectively) when compared to the poPSCs from control cultures (Figure 4C).

**Figure 5:**
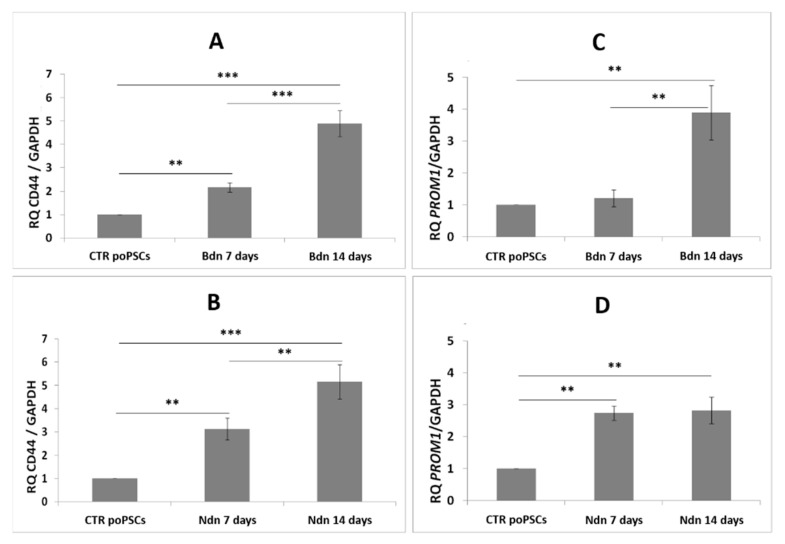
Expression of marker genes for CSCs: CD44 (**A**,**B**), PROM1 (**C**,**D**) at 7th and 14th day of culture in the presence of boldenone (**A**,**C**) and nandrolone (**B**,**D**) versus PSCs cultured without the addition of steroids at the transcript level as shown by RT-qPCR. The results (2^−ΔΔCt^) are presented as mean values with *n* = 3 ± standard deviation (SD). Statistical analysis: homogeneity of variance—Levene’s test, normality of distribution—Shapiro–Wilk test, one-way ANOVA and Tukey’s post-hoc test, ** *p* < 0.01; *** *p* < 0.001.

Analysis of cancer stem cells marker genes, CD44 and PROM1, by real time PCR showed their significant upregulation in both nandrolone or boldenone treated poPSCs, after 7 or 14 days of culture. CD44 mRNA expression in poPSCs cultured for 7 days in the presence of nandrolone was three times higher compared to the control cultures (** *p* < 0.01) (Figure 5B). After 14 days of nandrolone treatment, CD44 mRNA expression markedly increased (** *p* < 0.01) in comparison with the control cultures (Figure 5B). PROM1 mRNA expression in poPSCs cultured for 7 days in the presence of nandrolone was nearly three times higher (** *p* < 0.01) than that in the control poPSCs (Figure 5D). The expression of PROM1 mRNA after 14 days of poPSCs culture in the presence of nandrolone was unchanged compared to the 7-day nandrolone-exposed poPSCs cultures.

The level of CD44 expression in poPSCs cultured for 7 days in the presence of Bdn was nearly two-fold higher than in the control poPSCs (** *p* < 0.01). In turn, in poPSCs cultured for 14 days in the presence of Bdn, the level of CD44 expression increased nearly five-fold when compared to that in poPSCs control cultures (*** *p* < 0.001) (Figure 5A). There were no statistically significant differences in the expression level of PROM1 in poPSCs cultured for 7 days in the presence of boldenone compared to the control cultures (Figure 5C). On the other hand, the level of PROM1 expression in poPSCs cultured for 14 days in the presence of boldenone increased approximately four times compared to the control (** *p* < 0.01) (Figure 5C).

**Figure 6:**
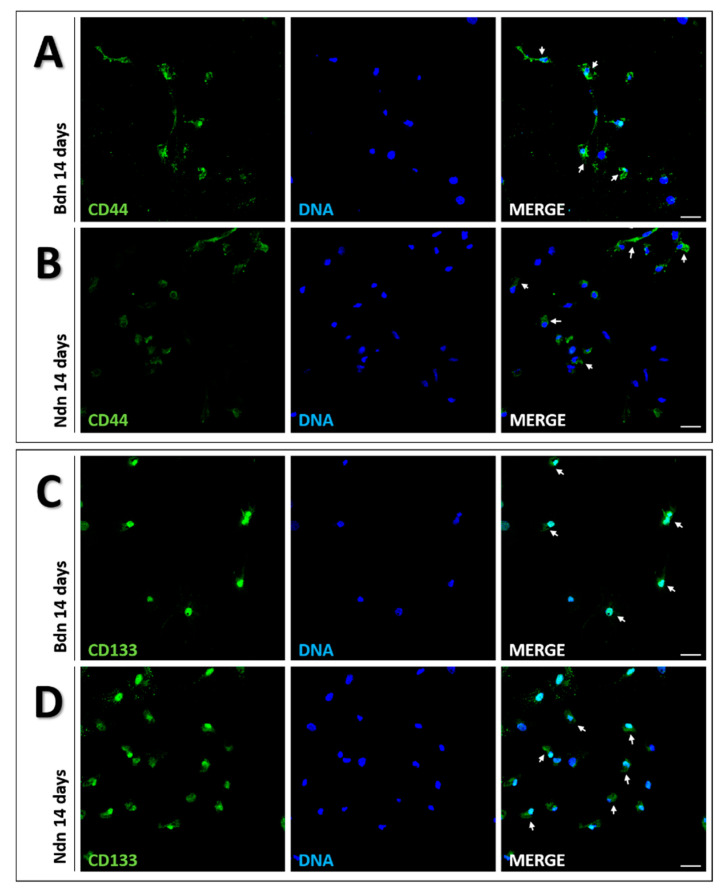
Immunofluorescent localization of CD44 (lines **A**,**B**) and CD133 (lines **C**,**D**) in poPSCs after 14 days of culture in the presence of boldenone at a dose of 100 µM (lines **A**,**C**) and nandrolone at a dose of 35 µM (lines **B**,**D**). Green signal—AlexaFluor 488 fluorescent dye, blue signal—DAPI, scale bars represent 100 µm. The immunofluorescence staining was repeated thrice; the figure shows the best representative micrographs selected from 3 replicates.

After 14-days of PSCs culture under the Ndn and Bdn influence, immunofluorescence analysis of CD44 and CD133—surface markers identifying a subset of cancer stem cells—was performed. Control poPSCs (data not shown) demonstrated no staining for these markers, but after 14 days of culture in the presence of Bdn (Figure 6AC) or Ndn (Figure 6 BD), the overall fluorescence intensity of CD44 and CD133 was markedly enhanced. The results of the IF analysis are consistent with those of the Western blot (WB) and quantitative real-time PCR analyses.

### 2.5. Oxygen Consumption in poPSCs after 7- and 14-Day In Vitro Culture in the Presence of Boldenone and Nandrolone

Since cell proliferation requires a bioenergetic support it was of interest whether nandrolone affected cell metabolism by impairing mitochondrial oxidative phosphorylation. To this aim, by using the Seahorse extracellular flux analyzer, mitochondrial oxygen consumption rate (OCR) and the extracellular acidification rate (ECAR) were simultaneously measured.

**Figure 7:**
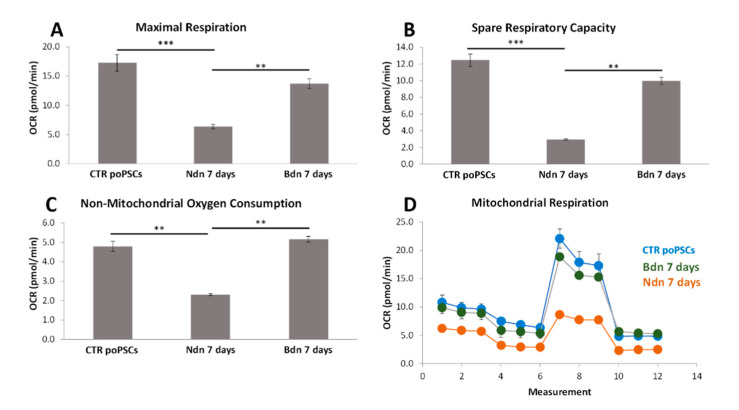
Seahorse XF Cell Mito Stress Test: The graphs show representative oxygen consumption rates (OCR) in mitochondrial respiration (**D**) broken down into maximal respiration (**A**), spare respiratory capacity (**B**), and non-mitochondrial oxygen consumption (**C**). Mitochondrial respiration was measured as OCR in poPSCs cultured for 7 days in the presence of nandrolone or boldenone. The obtained results were compared to the OCR level obtained on poPSCs cultured without the addition of these anabolic steroids (control). Results represent the mean with *n* = 4 ± standard deviation (SD). Statistical analysis: homogeneity of variance—Levene’s test, normality of distribution—Shapiro–Wilk test, one-way ANOVA, Tukey’s post-hoc test, ** *p* < 0.01; *** *p* < 0.001.

The Seahorse XF Cell Mito Stress Test showed that mitochondrial respiration (OCR) was significantly decreased in poPSCs cultured for 7 days in the presence of nandrolone compared to poPSCs cultured in the presence of boldenone or to control cultures (** *p* < 0.01 and *** *p* < 0.001, respectively) (Figure 7D). What is more, Ndn reduced the OCR level in the maximal respiration test (Figure 7A). Ndn significantly reduced also the OCR level in the spare respiratory capacity test (Figure 7B). The level of OCR in cells cultured for 7 days with the addition of nandrolone was four times lower than in the control and about three times lower compared to the boldenone test. It was also observed that after Ndn treatment, OCR level in non-mitochondrial oxygen consumption test was significantly decreased (** *p* < 0.01) (Figure 7C). The level of OCR in cells cultured for 7 days with the addition of nandrolone was about twice lower than in the control and about twice lower compared to the boldenone test. Interestingly, exposure to boldenone did not significantly affect the level of OCR relative to the control in any of the tests performed. The results obtained suggest that nandrolone may inhibit mitochondrial respiration and thus slowing poPSCs growth.

### 2.6. Nandrolone and Boldenone Affect the Expression and Phosphorylation of Proteins within the PI3K/Akt Pathway

**Figure 8:**
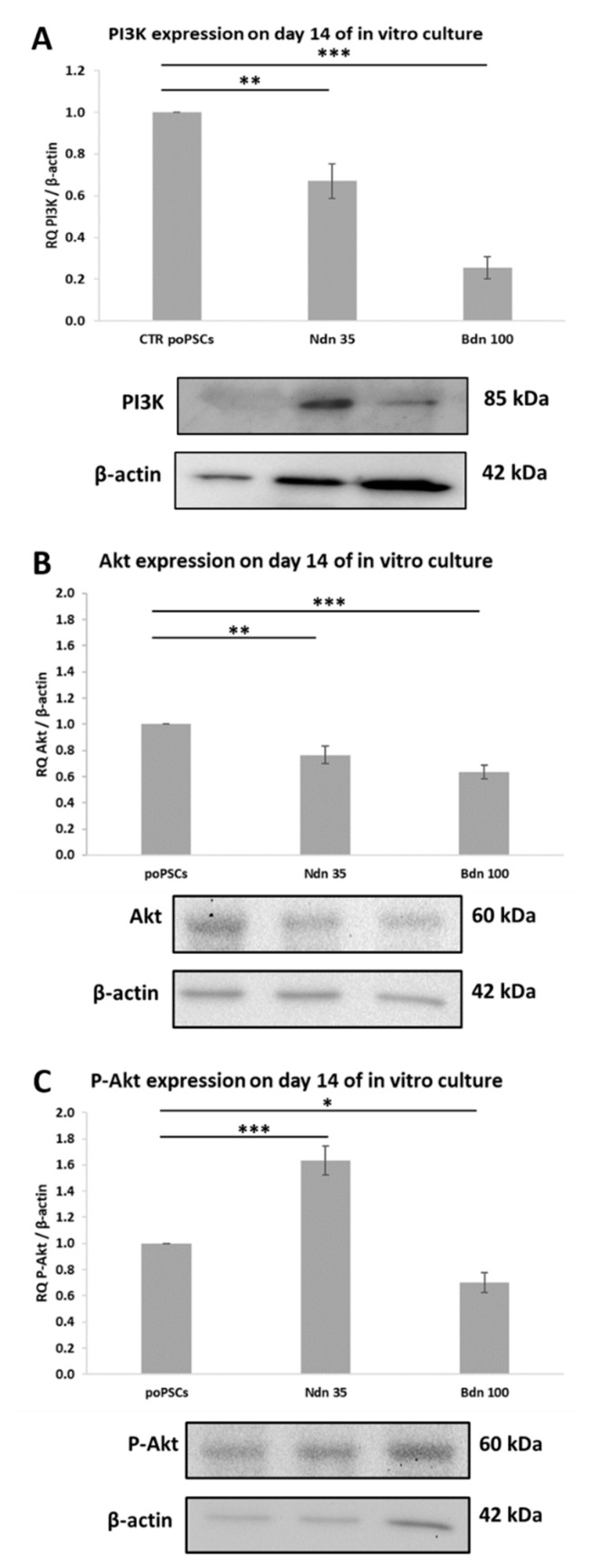
Expression of signaling pathway-related proteins: PI3K (**A**), Akt (**B**), and P-Akt (**C**) at the level of total protein after 14-Day culture in the presence of nandrolone and boldenone. The graphs depict the relative abundances (RAs) noticed for PI3K, Akt and P-Akt proteins obtained from measurements of the optical density of the bands representing a specific signal. Results represent the mean with *n* = 3 ± standard deviation (SD). Statistical analysis: homogeneity of variance—Levene’s test, normality of distribution—Shapiro–Wilk test, one-way ANOVA, Tukey’s post-hoc test, * *p* < 0.05; ** *p* < 0.01; *** *p* < 0.001.

After 14-days of poPSCs culture in the medium supplemented with nandrolone, the significant (** *p* < 0.01) decrease in the relative abundance (RA) of unphosphorylated Akt protein was observed (Figure 8B). In turn, the RA that has been determined for phosphorylated Akt (*p*-Akt) protein significantly increased (*** *p* < 0.001) after nandrolone treatment (Figure 8C). Simultaneously with the alterations identified for Akt, the significant decrease of PI3K (** *p* < 0.01) protein expression was confirmed following nandrolone treatment (Figure 8A). 

As has been shown in Figure 8, significant diminishments in the expression and phosphorylation of investigated proteins engaged in the PI3K/Akt pathway (* *p* < 0.05 or *** *p* < 0.001 for P-Akt or Akt and PI3K, respectively) were noticed after 14-Day culture of poPSCs in the medium enriched with boldenone.

## 3. Discussion

Beyond the deleterious macro-effects mentioned above, testosterone-derived anabolic steroids may affect directly cellular functions, acting together with either genetic or epigenetic factors determining their toxic, mutagenic, genotoxic, and carcinogenic results. This is possible because AAS exert their actions by several different mechanisms: (i) they can modulate androgen receptor expression and as a consequence intracellular metabolism; (ii) they can affect directly the androgen receptor and thus its subsequent interaction with co-activators and transcriptional activity; (iii) they can interfere with the glucocorticoid receptor expression eliciting an anti-catabolic effect; and (iv) they can function by the activation of non-genomic pathways [37]. In this context, the first aim of this study was to investigate whether poPSCs possess AR receptors through which Ndn and Bdn can affect them. In control poPSCs cultures, specific nuclear localization of the AR receptor has been demonstrated. This paved the way for experiments studying the effects of anabolic steroids on them. After 14-days of poPSCs culture in the presence of both nandrolone and boldenone, the specific nuclear localization of AR was found using immunofluorescence. The presence of AR in stem cells including poPSCs is not unusual. At the transcript level, AR was confirmed for the first time in undifferentiated ESCs by Chang’s team [38]. In turn, MSCs isolated from the bone marrow showing the AR receptor have already been used in regenerative medicine for the treatment of liver cirrhosis [39]. The AR receptors have also been found to regulate the progression of CSCs in various cancer types, including ovarian ones [40]. A study conducted by Chung et al. [41], using ovarian teratoma cells, provided evidence that ligand-independent AR functions in cancer stem/progenitor cells (CD133^+^ cells) facilitated ovarian teratoma cell growth. Moreover, Ling et al. [42] showed that AR expression promotes CSCs self-renewal through both classical androgen/AR activation and non-classical signaling pathways involving, inter alia, mTOR activation via the PI3K/Akt pathway. Based on the above-mentioned data and the results obtained in the current study, it might be possible that Ndn promotes the initiation of poPSCs neoplastic transformation by activating PI3K/Akt pathway. Our present investigation proved for the first time that a remarkable enhancement in Akt phosphorylation took place in nandrolone-treated poPSCs, whereas a declined semi-quantitative profile of Akt phosphorylation was recognized for boldenone-exposed poPSCs as compared to the control group of cell counterparts. Therefore, a decrease in the level of P-Akt that was markedly demonstrated after the use of boldenone suggests the inhibition of the PI3K/Akt pathway. In turn, an increase in Akt phosphorylation in nandrolone-treated poPSCs indicates an agonistic effect of this compound on the PI3K/Akt pathway. Interestingly, the qualitative and quantitative Western blot analysis showed that, in poPSCs treated with both nandrolone and boldenone, no increase in the protein level for PI3K was identified. A quite opposite effect of nandrolone was observed in MCF7 and MDA-MB-231 breast cancer cell lines, where nandrolone (at a concentration of 0.1 µM) inhibited their proliferation and migration by antagonizing the PI3K/Akt/NF-κB signaling pathway [43]. Masi et al. [43] provided the evidence that such nandrolone effect results from its binding to the membrane-bound receptor designated as oxo-eicosanoid receptor 1 (OXER1). OXER1 represents a novel link between androgens and their AR-independent action. Taking into consideration all the aforementioned findings, it should be stated that the activation of Akt under the influence of either hormones or testosterone-derived anabolic steroids is dose- and tissue-dependent. Moreover, it is worth highlighting that a broad spectrum of the non-classical and cell surface-dependent actions exerted by androgens are mediated by novel mARs, i.e., GPCRC6A, ZIP9/SLC39A9 and OXER1. Due to the fact that there is still not enough information regarding the molecular mechanisms underlying the intracellular events triggered by nandrolone and boldenone, further in-depth studies are needed. Taking into account the results of the semi-quantitative analysis of WB, slight changes in the expression of the AR and a decrease in the expression of PI3K/Akt kinases prove that nandrolone and boldenone act pleiotropically, probably activating different signal transduction pathways. This finding seems to confirm the broad-spectrum and multifaceted Ndn- and Bdn-dependent effects, which can justify a necessity for carrying out another more-detailed studies targeted at the exploration of other signaling pathways such as, e.g., those related to ERK1/2.

Nandrolone and boldenone, apart from desired effects, when used in very high doses by extended treatment periods, also cause adverse effects [44]. The AAS concentrations used in the present study (35 μM of nandrolone and 100 μM of boldenone) were chosen firstly, based on existing literature data, which have shown that micro-molar but not nano-molar concentrations cause significant adverse effects in cultured cells [45,46,47] and secondly based on the results from assessment of PCNA level in poPSCs cultured in the presence of different doses of Ndn or Bdn. The results presented herein proved that two of the five doses used of Ndn, 20 µM and 35 µM, produced a statistically significant suppression of poPSCs proliferation. Interestingly, although both doses that had been established at the level of either 35 µM for nandrolone or 100 µM for boldenone did not affect the viability and cytotoxicity estimated for the ex vivo-expanded poPSCs undergoing exposure to nandrolone, the onset of the scenario related to apoptotic cell death has been confirmed. Based on this and literature data, dose of 35 µM was used for further experiments. On the other hand, the lowest dose of Bdn that increased the proliferation rate of poPSCs was 100 μM, which was also used for further in vitro experiments. These concentrations might mimic the supraphysiological doses used by AAS-users but should not be interpreted as the actual concentration the organism is exposed to. The exposure time used in the study (7- and 14-days) was chosen to mimic repeated, prolonged treatment. The data from this relatively long exposure of poPSCs cannot be directly transferred to years of AAS abuse but could shed light on the molecular processes triggered by Ndn and Bdn. The results from the assessment of PCNA level presented herein are supported by similar findings reported by some investigators [48]. Similar to boldenone, other anabolic steroid boldione more than doubled PCNA expression in bovine large luteal cells cultured for 48 h in its presence. The authors of this study believe that the use of boldione may be the cause of granulomas observed in slaughtered calves in northern Italy, where this steroid is still often used [48]. While the effects of boldenone on the female reproductive system are not fully understood, numerous pathological changes have been observed in males. Groot and Biolatti [49] tested a group of bulls, in which the boldenone derivative designated as 17-β-boldenone was detected in the urine. Cysts in the prostate gland and excessive secretion of the prostatic fluid were observed in 45% of the bulls. In 70% of cases, there were abnormalities in testicular development and degenerative changes in the sperm-forming epithelium [49]. Given that the male reproductive system is heavily regulated by AR activity, the disorders observed by Groot and Biolatti [49] may have resulted from the action of boldenone via AR. The presented study was aimed to check whether boldenone, also acting through the AR receptor expressed in the poPSC, may contribute to their neoplastic transformation. In turn, the second of the tested AAS, nandrolone, despite its own anabolic nature, negatively affected the proliferation of neural stem cells in rats [50]. Nandrolone reduced proliferation of these cells in both males and females by acting through the cyclin-dependent kinase inhibitor-p21. A much stronger effect was observed in pregnant females, which indicates the involvement of estrogens in the action of nandrolone [50]. It is suggested that the high level of circulating estrogen in the blood during pregnancy in rats enhanced the effect of nandrolone, although the mechanism of this phenomenon is unknown. This is suspected to be related to the activation of relevant receptors during proliferation of neural stem cells and/or influencing various downstream signaling molecules. In addition, their studies have shown that the decrease in proliferation rate under the influence of nandrolone can be inhibited by the AR antagonist designated as flutamide [50]. Consistent with these findings are the results of the investigations by Agriesti et al. [29], in which the pharmacological dose of nandrolone significantly suppressed the proliferative activity of human hepatocarcinoma-derived cell lines (HepG2). A similar proliferation-inhibiting effect of nandrolone has been already reported on other cell lines such as the breast cancer cells [51] or rat Leydig R2C cells [52]. In turn, a quite opposite effect was documented by Chimento et al. [53]. Using the aforementioned rat Leydig R2C cell line, they showed that high doses of nandrolone administered together with peptide hormones like insulin-like growth factor-I (IGF-I), as it occurs in the doping practice, increased proliferation of rat Leydig R2C tumor cells via an estrogen-dependent mechanism. Taken together, the above presented data clearly indicate that the use of high doses of AAS causes an adverse effect; however, whether AAS enhance or significantly inhibit cell proliferation depends on the target site of action (i.e., whether they are somatic, stem, or cancer cells). In addition, the effect of their action directly depends what kind of molecular mechanism, estrogen- or androgen-dependent, they trigger. 

Inhibiting cell proliferation or slowing down their growth are generally linked to the modification of the cell metabolism because of the lower energy needs. To analyze this, we evaluated the bioenergetic metabolic fluxes in poPSCs, both control ones and those exposed to Ndn and Bdn for a long-term, by the Seahorse methodology. As expected, the mitochondrial oxygen consumption rate appeared to be lower in cells treated with AAS. These results suggest that nandrolone by inhibition of mitochondrial respiration slows poPSCs growth. Consistent with this observation are results obtained by Agriesti et al. [29]. Using HepG2 cell lines, these investigators showed that Ndn not only repressed mitochondrial respiration but also inhibited the respiratory chain complexes I and III and enhanced mitochondrial reactive oxygen species (ROS) production. Importantly, as previously reported, the illicit use of AAS, including Ndn and Bdn, is associated with serious adverse effects, including cellular neoplasmic transformation. Since the AR is expressed in a diverse range of tissues, AAS might be implicated in induction of their tumorigenesis [54,55]. Several studies demonstrated the key role of the androgen signaling in the regulation of normal or cancer stem cells (CSCs) [56,57]. CSCs are a small subgroup of neoplastic cells which are characterized by high self-renewal, extensive proliferation, and strong tumorigenesis capacity. The latter feature causes CSCs to play an important role in the genesis of various cancers [58,59]. A recent study revealed the relationship between androgens and hepatic CSCs maintenance, demonstrating that androgens and AR participated in their regulation through the NANOG-related pathway, a potent positive regulator of CSCs stemness [60].

Cancer stem cells are mostly identified by virtue of the expression of specific cell surface markers. Of these, two should be highlighted: CD44 and CD133, which are the most widely used markers in CSCs research [10]. More particularly, the expression of CD44 and CD133 distinguishes a number of cancer-initiating cells [20,61,62,63]. In our current investigation, a chronic exposure of poPSCs to pharmacological doses of both Ndn and Bdn has been shown for the first time to trigger the expression of such clusters of differentiation as CD44 and CD133, which indicates the risk of occurrence of molecular events characteristic for the neoplastic transformation of poPSCs. An increased expression of CD44 and CD133 following AAS exposure is the evidence of a phenotype shift, from poPSCs, which constitute the heterogeneous population of MSCs, to CSCs. This is a strong support of the current hypotheses that suggests that tumors originate from cells that carried out a process of “malignant reprogramming” driven by genetic and epigenetic alterations. Since CD133 transcription is controlled by both histone modifications and promoter methylation, expression of CD133 in ovarian cancer can be directly regulated by epigenetic modifications. The results reported here are in line with the notion that CD133 characterizes the ovarian tumor initiating cell population [64]. A meta-analysis of the relationship between CD133 expression, prognosis, and clinical and pathological features of ovarian cancer showed that, unlike CD44, high CD133 expression correlates with a worse prognosis in patients [65]. Moreover, based on a number of emerging evidence, as well as the results obtained, it can be concluded that cancer stem cells can switch their metabolic phenotypes in response to external stimuli for better survival [66]. Thanks to this, CSCs are more resistant to anti-tumor treatments than the non-stem cancer cells. Therefore, surviving CSCs might be responsible for metastasis and therapy resistance.

To sum up, our current investigation, which has been aimed at the development and optimization of the in vitro models for extrinsic anabolic steroid-dependent stimulation or modification applied to reprogram the molecular properties and cytophysiological functions of poPSCs, has proven that their exposure to either Ndn or Bdn brings about the enhancements in the expression of CSC-related markers. This may indicate that the approaches used for Ndn- or Bdn-assisted modulation of poPSCs are supposed to predominantly trigger their neoplastic transformation. On the one hand, molecular and epigenetic plasticity of isolated population of ovarian putative stem cells (oPSCs) offers a great opportunity for preclinical and clinical approaches targeted at ovarian cell/tissue engineering and surgical treatments based on regenerative and reconstructive medicine. These treatments can be mediated by oPSC-based auto-, iso-, allo-, or xenografting and are intended for infertile or sub-fertile female patients afflicted with ovary-specific dysfunctions/disorders such as polycystic ovary syndrome (PCOS). On the other hand, a variety of attributes related to augmented plasticity of oPSCs carry a considerable risk of initiating molecular pathways responsible for their oncogenic transformation (carcinogenesis). 

## 4. Materials and Methods

### 4.1. Sample Collection and poPSCs Isolation

Porcine ovaries were collected from sexually immature Polish Landrace gilts (approximately weighing 60 to 70 kg and 5 to 6 months of age) at a local abattoir under veterinarian control within 10 min of slaughter. Next, they were placed in sterile ice-cold Dulbecco’s modified phosphate-buffered saline (DPBS; pH 7.4, PAA The Cell Culture Company, Piscataway, NJ, USA) with the addition of antibiotics (Antibiotic/Antimycotic Solution; AASoln; 1% (*v*/*v*), PAA The Cell Culture Company) and taken to the laboratory within 1 h. After washing the experimental material twice using sterile DPBS, the ovarian cortex was separated from the ovarian cord with a scalpel and cut into uniform-size pieces of ~1 mm^3^ with a tissue slicer. The obtained fragments of ovarian cortex were subjected to a 2-h enzymatic digestion procedure in a Liberase™ TH Research Grade solution (0.26 U/mL in PBS; Sigma-Aldrich, St. Louis, MO, USA) in an incubator at temperature 37 °C, with 150 rotations/min. Next, enzymatic digestion was terminated by adding an equal volume of cold DPBS (+4 °C). After that, the resulting suspension was filtered through 100-, 70-, and 40-µm nylon strainers. In the further step, the cells were washed several times in sterile DPBS and recovered by centrifugation (90× *g* for 10 min). poPSCs were isolated by an immunomagnetic method, modified, and described by us previously [67], using a monoclonal antibody–anti-human SSEA-4, conjugated to magnetic beads (EasySepTM hESC/hiPSC SSEA-4 Positive Selection Kit, StemCell^TM^ Technologies, Vancouver, Canada). Next, the poPSCs were cultured in the maintenance medium (MM): DMEM/F12 medium (Sigma-Aldrich) supplemented with 2% B-27 (Thermo Fisher Scientific, Waltham, MA, USA) and 2 μL/mL SCF (Thermo Fisher Scientific). The prepared suspension of 3 × 10^3^ cells/mL was seeded into the culture dishes. Cells for total protein or total RNA extraction after the experiment were cultured in six-well polystyrene plates (Nunc™, Thermo Fisher Scientific) coated with poly-*L*-lysine (Sigma-Aldrich). Cells for immunofluorescence studies were cultured on eight-cell Lab-TekTM II-CC2 (Nunc™, Thermo Fisher Scientific) slides also coated with poly-*L*-lysine.

### 4.2. Evaluation of poPSCs Proliferation after 14-Day Exposure to Different Doses of Nandrolone or Boldenone

Following pre-culture, the medium was changed to fresh in 6-well plates (DMEM/F12 medium supplemented with 2% B-27 and 2 μL/mL SCF). For all in vitro experiments, Ndn (Sigma-Aldrich) and Bdn (Sigma-Aldrich) stocks were prepared in absolute dimethylsulfoxide (DMSO) and subsequently diluted in the culture medium. The final concentration of DMSO was kept at <5 μL/mL. poPSCs were exposed to different concentrations of Ndn (0-control, 15, 20, 25, 30, and 35 μM/L) or Bdn (0-control, 60, 80, 100, 120, and 140 μM/L). Cells in the presence of AAS were grown in the same conditions as in pre-cultures for 14 days. Every two days, the medium was changed, maintaining the dose regimen of both test compounds, and the cells were passaged when they reached 80% confluence. After completion of the culture, total protein was isolated from the cells growing in each well for subsequent semi-quantitative analysis of the PCNA proliferation marker.

### 4.3. poPSC Culture in the Presence of Selected Doses of Nandrolone or Boldenone

After preculture, the medium was changed to fresh (DMEM/F12, 2% B-27, 2 µL/mL SCF). The plates and slides were then divided into two equal subgroups. The first subgroup was given a boldenone solution in DMSO so as to obtain a concentration of 100 μM in the medium, and the second subgroup received a nandrolone solution to obtain a concentration of 35 μM in the medium. The Ndn and Bdn concentrations for the experiment were selected based on both: literature data and the results of the previous proliferation test. Every two days, the medium was changed, maintaining the dosing pattern of both test compounds, and the cells were passaged when they reached 80% confluence. After completion of culture on day 7 and 14, total protein and total RNA were isolated from cells growing in 6-well plates, and cells growing on eight-chamber slides were fixed for immunofluorescence.

### 4.4. ApoTox-Glo Triplex Assay

For apoptosis, viability, and cytotoxicity assays, poPSCs that had been cultured under the conditions of Ndn or Bdn supplementation were analyzed using the ApoTox-Glo Triplex Assay (Promega GmbH, High-Tech-Park, Mannheim, Germany) according to the manufacturer’s protocol. In brief, 20 µL of viability/cytotoxicity reagent mixture that was comprised of the permeable protease substrate known as glycylphenylalanyl-aminofluorocoumarin (GF-AFC) and a fluorogenic peptide substrate designated as bis-alanyl-alanyl-phenylalanyl-rhodamine 110 (bis-AAF-R110) was added to each well, and both of these compounds were shortly mixed by orbital shaking (at the parameters of 300 rpm and 30 s). The cells were subsequently incubated at 37 °C for 120 min in the presence of GF-AFC and bis-AAF-R110 reagent mixture. Fluorescence was measured at an excitation/absorption maximum wavelength of λ_ex_ equal to 400 nm and an emission maximum wavelength of λ_em_ equal to 505 nm (for assessment of cell viability) and λ_ex_/λ_em_ = 485 nm/520 nm (for evaluation of cytotoxicity) using a microplate spectrophotometer (Infinite M200; TECAN Group, Mannedorf, Switzerland). In the next step, 100 µL of Caspase-Glo 3/7 reagent was added to each well, and the samples were briefly mixed by orbital shaking (at the parameters of 300 rpm and 30 s) followed by incubation at room temperature (RT) for 120 min. Luminescence was quantitatively ascertained for 1 s according to the relevant protocol established for detection/determination of luminescence and its measurement was proportional to the amount of caspase activity present (Infinite M200, TECAN).

### 4.5. Immunofluorescence

Immunofluorescence, performed according to a technique developed and modified in our laboratory [67], was used to localize AR and cancer stem cells markers such as CD44 and CD133 in poPSCs incubated with steroids (boldenone and nadrolone). Additionally, PSCs cultured in the absence of steroids served as a control. After culture termination, cells were washed with PBS and fixed with cold 4% paraformaldehyde (PFA) in PBS for 10 min. After several washes with PBS, permeabilization of the cell membranes was performed by applying 0.1% Triton X-100 (Sigma-Aldrich) in Tris-buffered saline (TBS; pH 7.4). In the next step, nonspecific binding sites were blocked by an incubation with 5% normal goat serum (NGS, Sigma-Aldrich) in a humidified chamber for 40 min at room temperature. Then, NGS was removed, and the cells were incubated with the primary antibodies against the cancer stem cells markers CD44 (monoclonal mouse anti-CD44, ab6124, diluted 1:100, Abcam, Cambridge, UK) and CD133 (polyclonal rabbit anti-CD133, ab 19898, diluted 1:100, Abcam, Cambridge, UK) and against the androgen receptor-AR (polyclonal rabbit anti-AR, sc-816, diluted 1:50, Santa Cruz Biotechnology, Dallas, TX, USA) overnight at 4 °C in a humidified chamber. Subsequently, the cells were washed several times with TBST (TBS þ 0.1% Tween 20, Sigma-Aldrich) and incubated with the appropriate secondary antibody, either Alexa Fluor 488-labeled goat anti-rabbit (for AR and CD133) or goat anti-mouse (for CD44) at a 1:500 dilution (Thermo Fisher Scientific) for 1 h at room temperature in a dark, humidified chamber. Negative controls included cells incubated with 5% NGS. Immuno-labeled cells were mounted in VectaShield^®^ HardSet™ Mounting Medium with DAPI (Vector Laboratories, Burlingame, CA, USA), and they were analyzed with an OLYMPUS FV1200 FLUOVIEW scanning confocal laser microscope (parameters: OLYMPUS, Tokyo, Japan) under both 20× and 40× objective lenses.

### 4.6. Western Blot Analysis

Western blot analysis was performed according to a technique developed and modified in our laboratory [68]. Briefly, after termination of both poPSCs cultured in the presence of steroids for 7 or 14 days and poPSCs cultured without addition of steroids, they were washed twice with cold PBS. Next, total protein from all cultured cells samples was extracted using radioimmunoprecipitation assay buffer (RIPA; Thermo Scientific, Inc., Rockford, IL, USA) in the presence of protease inhibitor cocktail (Sigma-Aldrich). The suspension was then sonicated and centrifuged at 10,000× *g* for 20 min at 4 °C. The supernatant was collected and stored at −20 °C. The protein concentration was determined with the DC^TM^ Protein Assay (Bio-Rad Protein Assay; Bio-Rad Laboratories GmbH, München, Germany) using bovine serum albumin (BSA, Sigma-Aldrich) as a standard. Aliquots of cell lysates containing 30 mg of protein were solubilized in a sample buffer consisting of 62.5 mM Tris-HCl pH 6.8, 2% SDS, 25% glycerol, 0.01% bromophenol blue, and 5% β-mercaptoethanol (Bio-Rad Laboratories) and denatured at 99.9 °C for 3 min. After denaturation, the samples were separated via 10% (for AR, CD44, CD133, Akt, P-Akt, and PI3K) or 12% (for PCNA) sodium dodecyl-sulphate (SDS)-polyacrylamide gel electrophoresis (SDS-PAGE) under reducing conditions. The separated proteins were transferred onto a poly(vinylidene fluoride) (PVDF) membrane using a wet blotter in Genie Transfer Buffer (20 mM Tris, 150 mM glycine in 20% methanol, pH 8.4) for 90 min at a constant amperage of 350 mA. Then, the membranes were blocked with 5% non-fat milk in TBST (Tris-buffered saline with 0.1% *v/v* Tween20; Bioshop Inc., Burlington, VT, Canada) for 30 min at room temperature with gentle shaking, and next they were treated (overnight at ~4 °C) with the primary antibodies. The same primary antibodies as those used for immunofluorescent labelling were utilized as follows: immunoglobulins isotype G (IgGs) raised against AR (at a 1:200 dilution), against CD44 (at a 1:500 dilution), and against CD133 (at a 1:500 dilution). Additionally, IgGs against a proliferation marker PCNA (at a 1:1000 dilution) and IgGs against signaling pathway-related proteins such as: Akt (at a 1:1000 dilution), P-Akt (at a 1:1000 dilution), and PI3K (at a 1:1000 dilution) (Cell Signaling Technology; Danvers, MA, USA) were used. β-Actin was used as an internal control (monoclonal mouse anti-β-actin, diluted 1:2000; Sigma-Aldrich). The membranes were washed and incubated with an appropriate horseradish peroxidase (HRP)-conjugated secondary antibody (goat anti-mouse IgG for β-actin, CD44, and PCNA or goat anti-rabbit IgG for AR, CD133, Akt, P-Akt, and PI3K, Vector Laboratories; diluted 1:1000) for 1 h at RT. Immunoreactive protein bands were detected by chemiluminescence using Clarity™ Western ECL Blotting Substrate (Bio-Rad Laboratories). The blots were visualized using the ChemiDoc™, and all bands were quantified using the Image Lab™ 2.0 Software (Bio-Rad Laboratories). Semi-quantitative analysis was performed for three separately repeated experiments for each control and experimental group.

### 4.7. Total RNA Isolation and cDNA Synthesis

Total RNA was extracted from both poPSCs cultured in the presence of steroids for 7 or 14 days and poPSCs cultured without addition of steroids. Total cellular RNA was isolated using the EZ-10 Spin Column Total RNA Mini Preps Super Kit (Bio Basic Canada Inc.; Markham, ON, Canada) according to the manufacturer’s protocol. The quantity and quality of the total RNA were ascertained by measuring the absorbance at 260 and 280 nm with a NanoDrop ND2000 Spectrophotometer (Thermo Fisher Scientific; Wilmington, DE, USA). Moreover, RNA samples were electrophoresed on a 1% (wt/vol) denaturing agarose gel to verify the RNA quality and stored frozen at −80 °C. First-strand cDNA was prepared by reverse transcription (RT) using 1 mg of total RNA, random primers, and a High-Capacity cDNA Reverse Transcription Kit (Applied Biosystems; Foster City, CA, USA) according to the manufacturer’s protocol. The 20-mL total reaction volume contained random primers, dNTP mix, RNAse inhibitor, and Multi Scribe Reverse Transcriptase. RT was performed in a Veriti Thermal Cycler (Applied Biosystems) according to the following thermal profile: (1) 25 °C for 10 min, (2) 37 °C for 120 min, and (3) 85 °C for 5 min. Genomic DNA amplification contamination was checked using control experiments, in which reverse transcriptase was omitted during the RT step. The samples were kept at −20 °C until further analysis.

### 4.8. Quantitative Real-Time qPCR

The real-time PCR was performed according to the manufacturer’s protocol. For quantitative analysis, the mRNA levels of the investigated genes CD44 and PROM1 (CD133) in each sample were assessed using the TaqMan Gene Expression Assay (Applied Biosystems; assay ID: CD44 ARTZ9RP and PROM1 ARRWE6T). The level of glyceraldehyde-3-phosphate dehydrogenase (GAPDH; Applied Biosystems; assay ID: Ss03373286_u1) was estimated as an internal control. All real-time PCR experiments were performed in duplicate [68]. Amplifications were performed with a StepOne™ Real-Time PCR System (Applied Biosystems) according to the recommended cycling program (2 min at 50 °C, 10 min at 95 °C, 40 cycles of 15 s at 95 °C, and 1 min at 60 °C). Amplification of contaminating genomic DNA was checked by control experiments in which reverse transcriptase was omitted during the RT step. Threshold cycles (Ct values) for the expression of the investigated gene were calculated using StepOne software. All samples were normalized to GAPDH (ΔΔCt value). The relative expression of the genes of interest was expressed as 2^−ΔΔCt^ [69].

### 4.9. Seahorse Analysis

The cellular bioenergetics were determined using the XFp analyser (Agilent; Boston, MA, USA) kindly provided by Perlan Technologies (Warsaw, Poland). All assays were programmed (designed) in XF data acquisition Wave 2.6.1 software (Agilent, Boston, MA, USA). In each experiment, 3 baseline measurements were taken prior to the addition of any compound/substrate/inhibitor, and at least 3 response measurements were taken after the addition of each compound. Oxygen Consumption Rate (OCR) and Extracellular Acidification Rate (ECAR) were reported as absolute rates (pM/min for OCR and mpH/min for ECAR). While sensor cartridges were hydrated (overnight) and calibrated (XF Calibrant), cell plates were incubated in a 37 °C for 30 min prior to the start of an assay. All experiments were performed at 37 °C in non-CO_2_ conditions. Detailed protocols and their justification can be found at https://www.agilent.com/en/product/cell-analysis/how-torun-an-assay (accessed from June 2020 to May 2021). Additionally, detailed protocols were previously published [70,71,72].

#### Seahorse XF Measurement of ECAR and OCR Using Seahorse XF Cell Mito Stress Test

The pools/subpopulations of poPSCs, after 7- and 14-days of culture under Ndn or Bdn exposure were suspended in sterile (0.2-µm syringe strainer filtered) HBSS(+) w/o sodium bicarbonate (Gibco; Waltham, MA, USA) supplemented with 1 mM sodium pyruvate (Sigma–Aldrich, Saint Louis, MO, USA), 2 mM *L*-Glutamine (Sigma–Aldrich; Saint Louis, MO, USA), 10 mM *D*-glucose (Lonza Bioscience; Basel, Switzerland) and 5 mM HEPES (Sigma–Aldrich; Saint Louis, MO, USA) and adjusted to pH 7.4 with 0.1-N NaOH (Sigma–Aldrich; Saint Louis, MO, USA). Buffer factor of assay media was validated prior to experiments and was equal to 2.9 mM/pH. Next, cells were plated (4 × 10^5^ cells/well) in 180 µL on Agilent Seahorse 8-well XFp Cell Culture Miniplate and allowed to settle/adhere for 30 min at 37 °C. Real-time, noninvasive measurements of ECAR and OCR were obtained which correlated to acidification, mostly derived from glycolysis and mitochondrial function, respectively. Measurements were continued for 1 h and consisted of (i) a sample mixing time (each 1 min long) and (ii) a data acquisition period of 57 min. The latter consisted of 3 cycles with waiting time before each measurement lasting for 15 min.

### 4.10. Statistical Analysis

Statistical analysis was performed using Statistica 10.0 software (StatSoft, Inc.; Tulsa, OK, USA). For cell culture experiments, experiments were performed in quadruplicate (*n* = 5). Levene’s test for homogeneity of variance, the Shapiro–Wilk test for normality and one-way ANOVA followed by Tukey’s or Duncan’s post-hoc test were used to assess differences between control and experimental cultures. Western blot and real-time PCR analyses were repeated three times (in duplicate). The data are expressed as the mean ± SEM. Statistical significance was established at * *p* ≤ 0.05, ** *p* ≤ 0.01, and *** *p* ≤ 0.001.

## 5. Conclusions

The efforts undertaken in this study to comprehensively characterize molecular advantages of poPSCs and their potentially neoplastic cell derivatives are necessary to assess whether cell nuclei stemming from such NDCs will not fail to be epigenetically dedifferentiated in porcine SCNT-derived oocytes and resultant cloned embryos. It is noteworthy that these efforts have been conceptualized for the purpose of somatic cell cloning in pigs and different mammalian species for the first time. Furthermore, the approaches applied to sustainably ameliorate/repress pro-carcinogenic activity or eliminate oncogenicity (cancerogenicity) of ovarian MSC-like cells, the epigenomic memories and transcriptional profiles of which have been efficiently reprogrammed in porcine nuclear-transferred embryos, have not yet been devised. Therefore, optimizing these approaches is largely desirable for the needs of recognizing the suitability of ovarian putative stem cells that have undergone cancerous transformation to use them as NDCs for future studies aimed at SCNT in pigs and other mammalian species.

For the above-indicated reasons, thoroughly identifying factors that affect augmented epigenetic plasticity of the ovary-specific MSC-like cells and thereby enhanced reprogrammability of these NDCs in porcine nuclear-transferred embryos appears to be highly justified. As a consequence, this is of tremendous importance for the studies that attempt to improve the effectiveness of somatic cell cloning in pigs and a variety of mammalian species. The aforementioned scientific problems are also required to be widely resolved in order to remarkably increase the potential of practically using SCNT-based investigations for a broad spectrum of transgenic, biomedical, biopharmaceutical, and biotechnological research.

## Data Availability

Not applicable.

## Abbreviations

AAS	Anabolic androgenic steroids
AASoln	Antibiotic/antimycotic solution
Akt	A member of serine/threonine-specific protein kinase family (also known as protein kinase B; PKB) that plays a pivotal function in controlling the molecular balance between survival and death pathways in cells
AR	Androgen receptor
ASCs	Adult stem cells
Bdn	Boldenone
CSCs	Cancer stem cells
CD	Cluster of differentiation
ECAR	Extracellular acidification rate
ERK1/2	Extracellular signal-regulated protein kinases 1 and 2; also known as p44 mitogen-activated protein (MAP) kinase (a 44-kDa isoform of MAPK) and p42 mitogen-activated protein (MAP) kinase (a 42-kDa isoform of MAPK), respectively
ESCs	Embryonic stem cells
GPCRC6A	G protein-coupled receptor family C group 6 member A; a novel membrane androgen receptor (mAR) related to the extranuclear action of androgens
HA	Hyaluronic acid
HepG2	Human hepatocarcinoma-derived cell lines
IGF-I	Insulin-like growth factor-I
Klf-4	Krüppel-like factor-4 (also called gut-enriched Krüppel-like factor or GKLF); an evolutionarily conserved zinc finger-containing transcription factor that regulates diverse cellular processes such as cell growth, proliferation, differentiation, apoptosis, and somatic cell reprogramming
mARs	Novel membrane androgen receptors (unrelated to nuclear androgen receptors) that are engaged in a broad spectrum of non-classical, cell surface-initiated androgen actions
MSCs	Mesenchymal stem cells
mTOR	Mechanistic target of rapamycin (previously known as mammalian target of rapamycin) that represents a family of serine/threonine-specific protein kinases; mammalian target of rapamycin (mTOR) kinase that has been identified as a direct target of the rapamycin-FKBP12 (FK506-binding protein 12 kDa) complex; mTOR kinase is also designated as FK506-binding protein 12-rapamycin complex-associated protein 1 (FRAP1)
NANOG	Homeobox-containing transcription factor whose name stems from Celtic/Irish mythical word Tír na nÓg (i.e., Tir Na Nog; The Land of the Ever-Young)
NDCs	Nuclear donor cells
Ndn	Nandrolone
NF-κB	Nuclear factor-κB (nuclear factor kappa-light-chain-enhancer of activated B cells); a pleiotropic inducible transcription factor that occurs in almost all cell types and is the endpoint of a series of signal transduction events that are initiated by a vast array of stimuli related to many biological processes such as cytodifferentiation, cell growth, tumorigenesis, apoptosis, inflammation, and immunity
NOD-SCID	Non-obese diabetic/severe combined immunodeficient mouse model
OCICs	Ovarian cancer initiating cells
OCR	Oxygen consumption rate
Oct-4	Octamer-binding transcription factor-4 (also designated as POU5F1); a member of the family of POU (Pit-Oct-Unc)-domain and homeodomain transcription factors
oPSCs	Ovarian putative stem cells
OXER1	G protein-coupled oxo-eicosanoid receptor 1; a receptor of the arachidonic acid metabolite, i.e., 5-oxoeicosatetraenoic acid (5-oxoETE); known as a novel mAR involved in the rapid effects of androgens
PCDs	Potentially cancerous derivatives
PCNA	Proliferating cell nuclear antigen
PCOS	Polycystic ovary syndrome
PI3K	Phosphatidylinositol 3-kinase; a downstream kinase activated by receptor tyrosine kinases that generates a series of phosphorylated phosphoinositides, which recruit 3-phosphoinositide-dependent protein kinase-1 (PDPK1) activity to the plasma membrane, leading to activation of Akt
poPSCs	Porcine ovarian putative stem cells
RIPA	Radioimmunoprecipitation assay buffer
Rex1	Reduced expression gene 1 encoding a DNA-binding transcription factor known as reduced expression protein 1 or zinc finger protein 42 homolog
ROS	Reactive oxygen species
SCNT	Somatic cell nuclear transfer
Sox2	Sex-determining region Y (SRY)-box 2; a member of the high mobility group (HMG)-box family of DNA-binding transcription factors
ZIP9	Zinc transporter member 9; also designated as solute carrier family 39 member 9 (SLC39A9) or transmembrane zinc-influx transporter (Zrt)- and transmembrane iron-influx transporter (Irt)-like protein (ZIP) 9; represents both zinc (Zn^2+^)-iron (Fe^2+^) permease (ZIP) family and a novel membrane androgen receptor (mAR) family related to the extranuclear action of androgens
